# Supplementary material for: Differences in the Evolution of Clinical, Biochemical, and Hematological Indicators in Hospitalized Patients with COVID-19 According to Their Vaccination Scheme: A Cohort Study in One of the World’s Highest Hospital Mortality Populations
Source: Vaccines (Basel). 2024 Jan 11;12(1):72. doi: 10.3390/vaccines12010072 (PMC10821037; doi:10.3390/vaccines12010072)
Supplement: Supplementary file 1 [file vaccines-12-00072-s001.zip › vaccines-2773952-supplementary.pdf]

Table S1. Cutoff scores, Area under the curve (AUC), sensitivity, and specificity for various clinical measures examined in this study.

Variable	Group	AUC	S.E.	P	95%CI		Cutoff	Sens	Esp
Arterial pH	Not vac	0.25	0.02	<0.001	0.22	0.28	7.2305	0.71	0.97
	Astra	0.29	0.04	<0.001	0.21	0.37	7.3100	0.62	0.83
	Pfizer	0.37	0.03	<0.001	0.31	0.43	7.3005	0.67	0.89
AST	Not vac	0.60	0.02	<0.001	0.56	0.63	44.2	0.38	0.26
	Astra	0.51	0.04	0.806	0.43	0.59	34.3	0.49	0.52
	Pfizer	0.66	0.03	<0.001	0.60	0.73	33.4	0.55	0.32
ALT	Not vac	0.46	0.02	0.030	0.43	0.50	42.0	0.30	0.39
	Astra	0.48	0.04	0.614	0.40	0.56	27.5	0.42	0.54
	Pfizer	0.58	0.04	0.035	0.51	0.65	28.1	0.48	0.35
ALP	Not vac	0.55	0.02	0.009	0.51	0.59	81.2	0.48	0.37
	Astra	0.41	0.05	0.056	0.32	0.51	94.4	0.55	0.59
	Pfizer	0.69	0.04	<0.001	0.62	0.76	84.1	0.64	0.29
Platelets	Not vac	0.36	0.01	<0.001	0.34	0.39	291500.0	0.31	0.51
	Astra	0.48	0.03	0.517	0.43	0.54	264500.0	0.43	0.49
	Pfizer	0.48	0.02	0.340	0.43	0.52	267500.0	0.46	0.48
MPV	Not vac	0.62	0.01	<0.001	0.59	0.64	11.1	0.40	0.24
	Astra	0.51	0.03	0.761	0.45	0.57	11.0	0.28	0.33
	Pfizer	0.65	0.02	<0.001	0.60	0.70	11.5	0.34	0.18
PLR	Not vac	0.42	0.02	<0.001	0.40	0.45	450.8	0.46	0.55
	Astra	0.50	0.03	0.995	0.44	0.56	319.4	0.44	0.49
	Pfizer	0.46	0.03	0.129	0.41	0.51	284.2	0.46	0.55
ESR	Not vac	0.45	0.03	0.073	0.40	0.50	28.5	0.58	0.62
	Astra	0.59	0.08	0.231	0.44	0.74	28.0	0.62	0.36
	Pfizer	0.57	0.06	0.216	0.46	0.69	30.5	0.42	0.38

AUC: Area under the curve. S.E.: Standard error. CI: Confidence intervals. Sens: Sensitivity. Spec: Specificity. Not vac: Not vaccinated. Astra: Oxford–AstraZeneca. Pfizer: Pfizer–BioNTech. ALP: alkaline phosphatase, expressed in international units per liter (IU/L). AST: aspartate amino transferase, expressed in IU/L. ALT: alanine aminotransferase, expressed in IU/L. Platelets X 1000, expressed in cells per microliter of blood. MPV: mean platelet volume expressed in femtolitre. PLR: Platelet-to-lymphocyte ratio. ESR: Erythrocyte Sedimentation Rate, mm/hour.
